# Whole-genome sequencing of spermatocytic tumors provides insights into the mutational processes operating in the male germline

**DOI:** 10.1371/journal.pone.0178169

**Published:** 2017-05-22

**Authors:** Eleni Giannoulatou, Geoffrey J. Maher, Zhihao Ding, Ad J. M. Gillis, Lambert C. J. Dorssers, Alexander Hoischen, Ewa Rajpert-De Meyts, WGS500 Consortium, Gilean McVean, Andrew O. M. Wilkie, Leendert H. J. Looijenga, Anne Goriely

**Affiliations:** 1 Clinical Genetics Group, MRC-Weatherall Institute of Molecular Medicine, University of Oxford, Oxford, United Kingdom; 2 Nuffield Division of Clinical Laboratory Sciences, Radcliffe Department of Medicine, University of Oxford, Oxford, United Kingdom; 3 Department of Pathology, Erasmus MC—University Medical Center Rotterdam, Rotterdam, The Netherlands; 4 Department of Human Genetics, Radboud University Medical Center, Nijmegen, The Netherlands; 5 Department of Growth & Reproduction, Copenhagen University Hospital (Rigshospitalet), Copenhagen, Denmark; 6 Wellcome Trust Centre for Human Genetics, University of Oxford, Oxford, United Kingdom; Stanford University School of Medicine, UNITED STATES

## Abstract

Adult male germline stem cells (spermatogonia) proliferate by mitosis and, after puberty, generate spermatocytes that undertake meiosis to produce haploid spermatozoa. Germ cells are under evolutionary constraint to curtail mutations and maintain genome integrity. Despite constant turnover, spermatogonia very rarely form tumors, so-called spermatocytic tumors (SpT). In line with the previous identification of *FGFR3* and *HRAS* selfish mutations in a subset of cases, candidate gene screening of 29 SpTs identified an oncogenic *NRAS* mutation in two cases. To gain insights in the etiology of SpT and into properties of the male germline, we performed whole-genome sequencing of five tumors (4/5 with matched normal tissue). The acquired single nucleotide variant load was extremely low (~0.2 per Mb), with an average of 6 (2–9) non-synonymous variants per tumor, none of which is likely to be oncogenic. The observed mutational signature of SpTs is strikingly similar to that of germline *de novo* mutations, mostly involving C>T transitions with a significant enrichment in the ACG trinucleotide context. The tumors exhibited extensive aneuploidy (50–99 autosomes/tumor) involving whole-chromosomes, with recurrent gains of chr9 and chr20 and loss of chr7, suggesting that aneuploidy itself represents the initiating oncogenic event. We propose that SpT etiology recapitulates the unique properties of male germ cells; because of evolutionary constraints to maintain low point mutation rate, rare tumorigenic driver events are caused by a combination of gene imbalance mediated via whole-chromosome aneuploidy. Finally, we propose a general framework of male germ cell tumor pathology that accounts for their mutational landscape, timing and cellular origin.

## Introduction

Spermatocytic tumor (SpT; previously known as spermatocytic seminoma, also referred to as TGCT type III) is a rare testicular germ cell tumor (TGCT) that is distinct epidemiologically and pathologically from the more common classical seminoma and non-seminoma that occur in adolescents and young men [1,2]. SpT presents as a slow growing, often large (3–30 cm) but well-circumscribed tumor characterized histologically by the presence of three cell types that resemble cells observed in normal adult spermatogenesis: a large cell measuring ~50–100 μm in diameter and resembling spermatocytes, which explains the origin of the tumor’s name; a lymphocyte-like small cell (~6–8 μm in diameter) and a more common intermediate cell-type (~15–20 μm). These tumors are restricted to the testis and have no ovarian equivalent. Although TGCTs are the most frequent tumors among Caucasian men aged 15–44 years in the US, occurring at a rate of 5–7 cases per 100,000 men [3,4], SpT only represent 0.6–2% of all diagnosed TGCTs, corresponding to a reported incidence of 0.4–2 cases per 1,000,000 [5]. Moreover, SpT is reported as being more prevalent in older men, with a mean age at diagnosis of 54 years, although the diagnostic age range is wide (19–92 years) [6]. Clinically, the vast majority of these uncommon tumors have an indolent course and orchidectomy is generally curative; however rare occurrences of sarcomatous transformation and metastasis associated with aggressive behavior and poor prognosis have been reported [2,7].

Interestingly, while classical type II TGCTs, now referred to as GCNIS (germ cell neoplasia in situ)-related TGCT [2], originate from developmentally arrested embryonic germ cells (gonocytes) and develop through the precursor GCNIS (previously known as carcinoma in situ or intratubular germ cell neoplasia, unclassified) [3,4,8], SpT represents a more differentiated testicular neoplasm derived from adult progenitors, which explains the older mean age at diagnosis and the lack of an ovarian equivalent [9]. Spermatogenesis is a highly regulated process that requires, from puberty onwards, the cyclic turnover of spermatogonial stem cells to generate millions of haploid spermatozoa every day. In humans, this activity is initiated when primordial germ cells (PGCs), derived from the inner cell mass, migrate and reach the developing bipotential gonads at gestation week 5, where specific patterns of gene expression in somatic cells stimulate either male or female development. The commitment to male development, triggered by the expression of the Y chromosome-linked *SRY* gene, involves the down-regulation of genes required for initiation of meiotic replication and entry into meiotic prophase I. In this setting, PGCs, now termed gonocytes, begin to multiply rapidly. At 17–18 weeks of gestation, gonocytes begin to mature into pre-/fetal spermatogonia, a process involving down-regulation of pluripotency factors, gradual migration to the basal lamina of the sex cords, and a relative quiescence until after birth [10,11]. Following testicular descent at or around birth, a surge in testosterone production and other testicular hormones occurs [12]. It is believed that during this period, sometimes referred to as “mini-puberty”, the remaining neonatal gonocytes migrate to the periphery of the cord and mature into type-A spermatogonia. By the age of 2 years at the latest, all gonocytes have either differentiated or have been eliminated by apoptosis. This mini-puberty step is essential for germ cell proliferation and differentiation later in life because a failure to complete this stage, caused for example by cryptorchidism, results in loss of germ cells and increased risk of infertility [13]. During early childhood (around 3–4 years of age), a few type-A spermatogonia may mature to form type-B spermatogonia and occasionally primary spermatocytes, although these do not complete meiosis and die. At puberty, spermatogenesis ‘*sensu stricto*’ begins when spermatogonial stem cells enter a regular pattern of mitoses and meioses occurring in synchrony with the epithelial cycle (i.e. every 16 days) that support both self-renewal and differentiation into spermatozoa during adulthood.

Whilst it is well accepted that unlike classical type II TGCTs that originate during embryogenesis, SpTs derive from post-natal precursors, the exact nature of the cell of origin of this tumour has been controversial; despite its name, SpT is now thought to derive from spermatogonial cell populations. For example, cytofluorimetric analyses of DNA content have failed to show the presence of a haploid component, and mitotic figures are frequently seen in all three cell populations [14,15]. These observations suggest that SpTs occur through neoplastic transformation of pre-meiotic germ cells, probably at a transition stage between spermatogonia and spermatocytes [16]. Moreover, immunohistochemistry studies concur that distinct sub-classes of SpTs may exist, each characterized by the expression of different combinations of protein markers [17,18,19], raising the possibility that SpTs are not a single entity but represent a heterogeneous tumor type with multiple cellular and/or developmental time origins.

Although the mechanisms leading to the occurrence of SpT formation have not been pinpointed so far, we previously proposed that a subset of these tumors represent the extreme and rare outcome of a universal process termed selfish spermatogonial selection that takes place in the testis of all men as they age [20]. In this process, rare spontaneously-arising “selfish” gain-of-function mutations in spermatogonia confer a growth/survival advantage, leading to clonal expansion of the mutant spermatogonial cells over time. Unlike classical somatic mutations, mutations arising in germ cells are heritable: selfish mutations are associated with a higher risk of transmission to the next generation than neutral *de novo* mutations and account for the high spontaneous birth rate and paternal age effect of some severe congenital disorders [21]. All selfish mutations documented so far affect proteins acting in the receptor tyrosine kinase (RTK)/RAS/MAPK pathway. Targeted sequencing of genes in this pathway in a panel of 55 SpTs showed that ~20% carried activating mutations in either *FGFR3* (2 samples) or *HRAS* (7 samples). Strikingly, all SpTs carrying selfish mutations were diagnosed in significantly older men [average: 76.1 yr (range: 67–87 yr) vs. 55.3 yr (range: 33–86.5 yr) for mutation-negative samples] [22].

Being rare and associated with a good prognosis, SpTs may be considered of little clinical importance. However, biologically, these tumors represent a unique model for the study of cellular processes specific to the post-natal male germline including regulation of spermatogenesis, mitosis-meiosis transition and, paradoxically perhaps, the occurrence of *de novo* germline mutations. Here, in order to gain further insights into the homeostatic properties of male germ cells and the origin and associated pathogenesis of SpTs, we have extended our targeted sequencing of SpT cases and performed whole-genome sequencing of four tumor and matched normal pairs and a SpT singleton, to our knowledge the only existing collection of frozen tumor and matched normal samples for this rare tumor type. We show that the tumors we sequenced are characterized by very low point mutation rates and exhibit signatures typical of germline *de novo* mutations, highlighting the unique cellular context of their tissue of origin. Our genome-wide analysis suggests that the SpTs we sequenced have arisen through an unusual mutational mechanism whereby tumor growth is driven by a specific assortment of whole-chromosome gains and losses. This process may be related to a failure to complete the mitosis-meiosis transition, a cellular process occurring post-natally only in male germ cells.

## Results

### Genetic and epidemiological heterogeneity of SpTs

**Fig 1:**
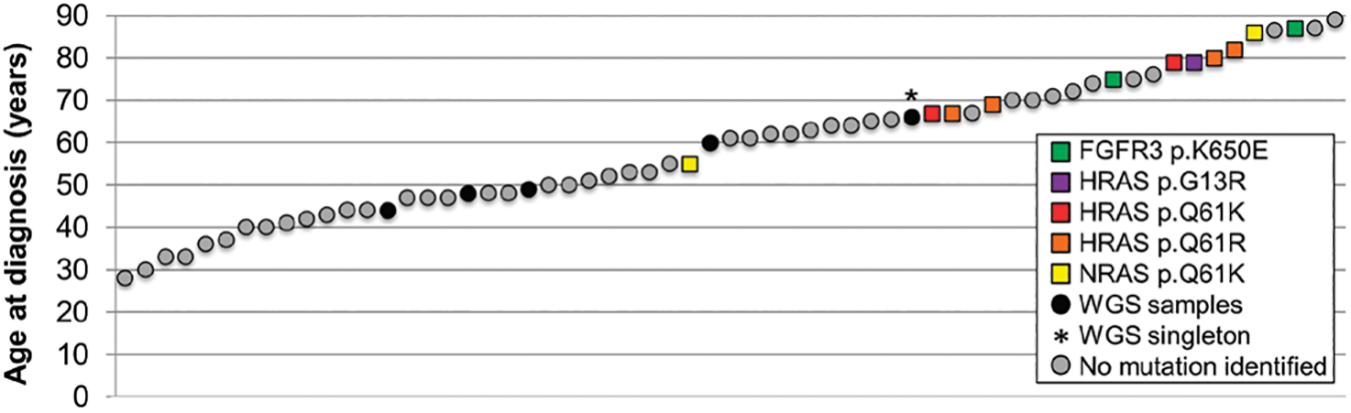
Mutation status of SpTs in relation to age at diagnosis. Age at presentation of 61 SpT samples that were screened for hotspot mutations in genes associated with selfish spermatogonial selection in this and in previous studies [17, 18]. Mutational status indicated by color chart on the Figure. In this study, out of the 29 FFPE samples screened (S1 Table), NRAS p.Q61R mutations were identified in two cases (aged 55 and 86 years); the other mutation-positive cases were previously documented in [17, 18].

We previously established that a subset of SpTs carry pathogenic ‘selfish’ mutations in components of the RTK/RAS/MAPK cascade, the signaling pathway known to be dysregulated in selfish spermatogonial selection and paternal age effect (PAE) disorders [22] (Fig 1). To further assess the contribution of selfish mutations to SpTs, a panel of 29 archival SpT samples (23 of which had previously undergone screening for a limited number of mutations [22,23]) were analyzed for the presence of mutations at hotspot regions in seven genes for which germline mutations have been implicated in selfish spermatogonial selection (*FGFR2*, *FGFR3*, *PTPN11*, *RET*, *HRAS*, *KRAS* and *NRAS)* [20,24] using molecular inversion probes (MIPs) and Ion PGM sequencing (S1 and S6 Tables). Because DNA extracted from formalin-fixed paraffin embedded (FFPE) archival material is often degraded and of poor quality, MIPs were designed to capture short (60–120 bp) genomic sequences (see Methods). Two tumors (H8T and SS8 from individuals aged 55 and 86 years respectively) harbored the same heterozygous oncogenic mutation in *NRAS* (c.182A>G, encoding p.Q61R), which was present in 47% (115/245) reads in H8T and in 38% (48/128) reads in SS8 (Fig 1 and S1 Fig). NRAS p.Q61R is a well-known oncogenic substitution previously reported in > 1300 tumor samples (COSMIC) including skin, thyroid, large intestine and hematological malignancies. Although NRAS p.Q61R has never been reported in the germline, substitutions associated with weaker gain-of-function such as p.G13D and the non-canonical p.I24N/L, p.T50I and p.G60E have been associated with the congenital disorder Noonan syndrome [25]. No mutations at known hotspots were present in the other samples (S1 Table). To date, oncogenic mutations in three genes (*FGFR3*, *HRAS*, *NRAS*) have been detected in 11 of the 61 (18%) SpTs that have been screened molecularly for RTK/RAS mutational hotspots and for which we possess information about the age of excision (Fig 1). All mutation-positive samples were from patients ≥ 55 years old and were significantly older than samples without detected mutation (mean 75.1 years *vs*. 55.3 years in mutation-negative samples; t-test p < 0.0001).

### Whole-genome landscape of SpTs: Ploidy, zygosity, CNVs and rearrangements

**Fig 2:**
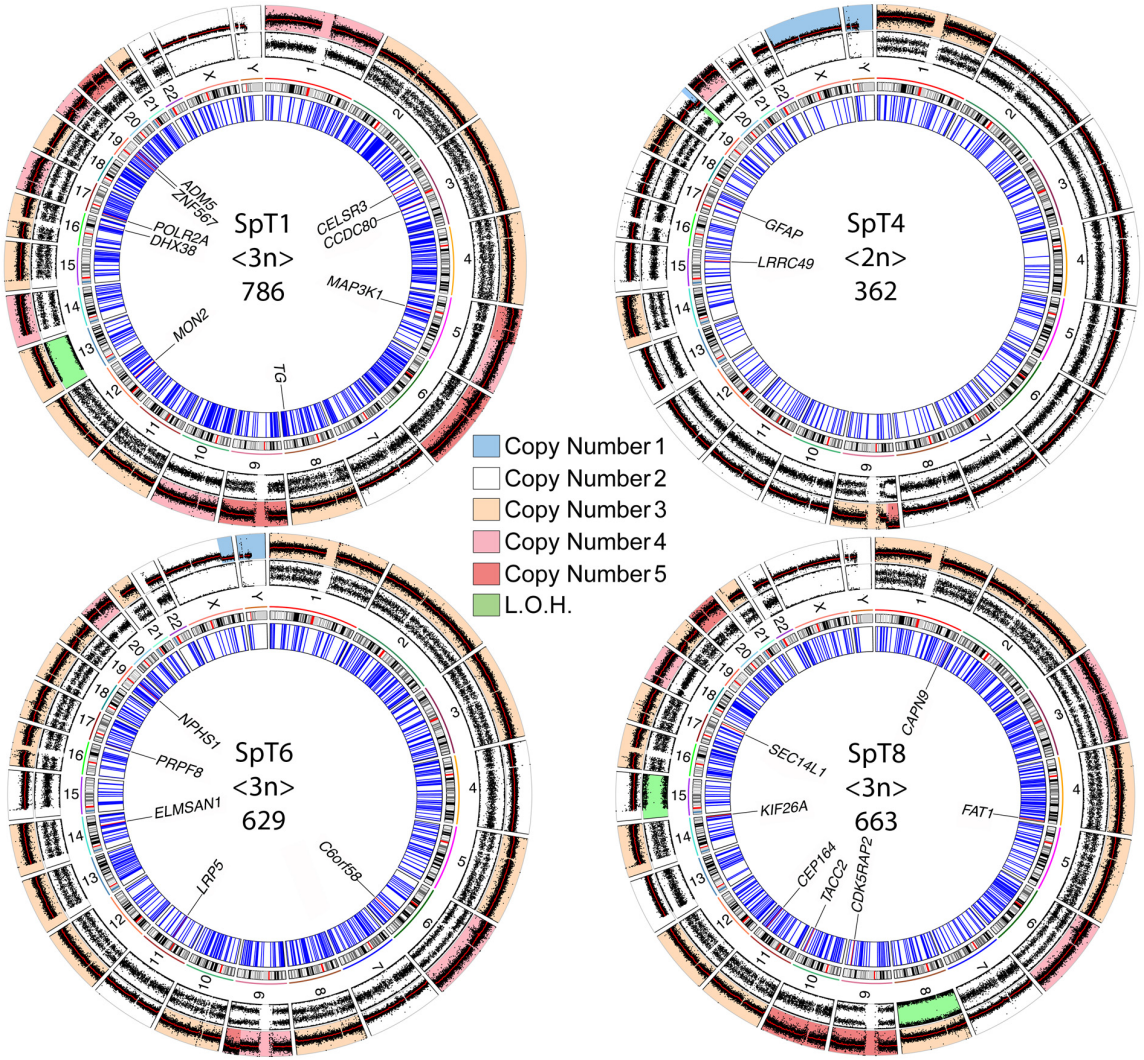
Circos plots of the four SpT tumors with matched normal tissue showing somatically acquired events. From outside inwards, the first ring represents chromosomal copy number (inferred from relative sequencing depth); color indicates chromosomal copy number as illustrated in key; the second ring shows the relative allele frequency of the minor (B-allele) allele for one million common SNPs (green color indicates LOH (loss of heterozygosity)); the third ring indicates chromosome number adjacent to a representative ideogram of the chromosome, with centromeric region highlighted in red. In the inner ring, radial blue lines correspond to all called acquired SNVs and indels, with validated coding non-synonymous mutations shown as radial red bars and labelled by their respective gene name. The tumor name, ploidy number (also see Table 1 and S2 Table) and total number of SNVs and indels (S3 Table) are presented in the center of each plot. Details of the location of the coding variants are given in S3A Table.

To gain further insights into the pathogenesis of SpTs, we sequenced the whole-genomes of four fresh frozen (FF) tumor-matched normal (blood or tissue adjacent to the tumor) pairs (SpT1, SpT4, SpT6, SpT8) sampled from individuals aged 44–60 years and a FF tumor singleton (SpT3, from a man aged 66 years), all of which had previously tested mutation-negative in our targeted resequencing screen [22,23] (Fig 1 and S1 Table). Tumors and matched-normal controls were sequenced using Illumina technology to a mean coverage of 52x and 26x, respectively. We determined the chromosomal copy number of each tumor based on relative coverage depth of the tumor to its matched diploid control—or reference diploid genome in the case of the SpT3 singleton (Fig 2, S2 Fig and S2 Table). The contamination of tumor DNA by normal diploid cells was shown to be minimal for most tumors, except for SpT8 that exhibited an estimated 15–20% wild-type contamination. The median autosome number was 72 (range: 50–99), confirming the extensive aneuploidy previously described for these tumors [15,26,27] (Table 1). One tumor was near-tetraploid [SpT3 (99 autosomes and 2 copies each of X and Y)], three tumors were near-triploid [SpT1 (76 autosomes and 2 copies each of X and Y), SpT6 (64 autosomes, 2 copies of X and 1 copy of Y), SpT8 (72 autosomes and 2 copies each of X and Y)], and one tumor was near-diploid [SpT4 (50 autosomes and 1 copy of X and Y)]. For tumor SpT1, previous analyses of single cell karyotypes (performed by fluorescence in situ hybridization (FISH) and spectral karyotyping (SKY)) are in agreement with the chromosome number obtained from relative coverage depth of the WGS data [15,27]. Hence, sequence data generated by bulk tumor DNA analysis reflect the integral chromosomal composition of the tumor.

**Table 1:** Chromosome Copy number and zygosity of whole-genome sequenced SpT samples.

Chr	SpT1 chr number	SpT1 zygosity	SpT3 chr number	SpT3 zygosity	SpT4 chr number	SpT4 zygosity	SpT6 chr number	SpT6 zygosity	SpT8 chr number	SpT8 zygosity	adjusted P-value loss	adjusted P-value gain
**1**	4	2:2	4	2:2	3	2:1	3	2:1	3	2:1	NS	NS
**2**	3	2:1	4	2:2	2	1:1	3	2:1	3	2:1	NS	NS
**3**	3	2:1	4	2:2	2	1:1	3	2:1	4	3:1	NS	NS
**4**	3	2:1	4	2:2	2	1:1	2	1:1	3	2:1	NS	NS
**5**	4 (5)#	3:1 (3:2)	4	2:2	2	1:1	3	2:1	3	2:1	NS	NS
**6**	5	3:2	6	5:1	2	1:1	4	3:1	4	3:1	NS	NS
**7**	2	1:1	5	3:2	2	1:1	2	1:1	2	1:1	**0.047**	NS
**8**	3	2:1	3	2:1	2	1:1	3	2:1	3	3:0	NS	NS
**9**	5	3:2	6	4:2	3 (4–5)#	2:1 (3:1–4:1)	4 (5)#	3:1 (4:1)	5	4:1	NS	**0.0097**
**10**	4	2:2	5	3:2	2	1:1	3	2:1	5	3:2	NS	NS
**11**	3	2:1	4	2:2	2	1:1	2	1:1	3	2:1	NS	NS
**12**	3	2:1	4	2:2	2	1:1	3	2:1	3	2:1	NS	NS
**13**	3	3:0	3	2:1	2	1:1	3	2:1	2	1:1	**0.047**	NS
**14**	4	3:1	3	2:1	3	2:1	3	2:1	3	2:1	NS	NS
**15**	3	2:1	3	2:1	2	1:1	2	1:1	2	2:0	**0.0066**	NS
**16**	3	2:1	4	2:2	2	1:1	3	2:1	3	2:1	NS	NS
**17**	4	3:1	4	2:2	2	1:1	3	2:1	3	2:1	NS	NS
**18**	3	2:1	4	2:2	3	2:1	3	2:1	4	2:2	NS	NS
**19**	4	3:1	5	3:2	2	1:1	3	2:1	3	2:1	NS	NS
**20**	5	3:2	8	6:2	4	2:2	4	2:2	5	3:2	NS	**< 1e-05**
**21**	3	2:1	8	6:2	2	1:1	3	2:1	4	3:1	NS	NS
**22**	2	1:1	4	2:2	2	1:1	2	1:1	2	1:1	**0.0066**	NS
**X**	2	2:0	2	2:0	1	1:0	2	2:0	2	2:0	NA	NA
**Y**	2	2:0	2	2:0	1	1:0	1	1:0	2	2:0	NA	NA

# in cases of chromosomal arm amplifications, the statistical tests (see S2A Table) were run using the lowest chromosome copy number (full integral chromosome copy number)—detail of breakpoints are given in S2B Table; NS (not significant); NA (not applicable).

Overall the tumors harbored more chromosomal gains than losses over their basic ploidy number (Fig 2, S2 Fig, Table 1 and S2A Table). Across the five tumors, we observed consistent gains for chr9 (Permutation Test, adjusted p-value for multiple testing = 0.0097) and chr20 (p < 1x10^-5^) as well as recurrent losses of chr15 and chr22 (p-value = 0.0066), and chr7 and chr13 (p-value = 0.047) over the basic ploidy number of each tumor. Except for a few chromosomal arms [gains of chr5p (which was present at 5 copies in SpT1, a tumor with 4 whole chr5), chr9p (5 copies in SpT4, a tumor with 3 whole chr9), and chr9q (5 copies in SpT6, a tumor with 4 whole chr9) (S3 Fig), and losses of chr19q in SpT4 and chrXq in SpT6; (S2A Table; for breakpoint details see S2B Table)], the observed copy number variations involve whole-chromosomes. No acquired structural rearrangements, large (> 100 kb) intra-chromosomal copy number variations or gene fusions were observed. Analysis of the relative coverage depth for the four tumors with their matched controls on a gene-by-gene basis (exon by exon) was performed and no local amplification or intra-chromosomal copy number variations were observed. Analysis using the model-based algorithm OncoSNP-Seq [28], further confirmed the copy number and zygosity (B-allele frequency) determined for each chromosome using the relative allelic ratio of common SNPs (Fig 2, S2 Fig and S2B Table). We observed occurrences of loss of heterozygosity (LOH) for whole-chromosomes, equivalent to uniparental disomy (UPD) or trisomy, for chr13 (SpT1, allelic ratio 3:0), chr8 (SpT8, allelic ratio 3:0) and chr15 (SpT8, allelic ratio 2:0).

### Spectrum of somatic mutations in SpTs

**Fig 3:**
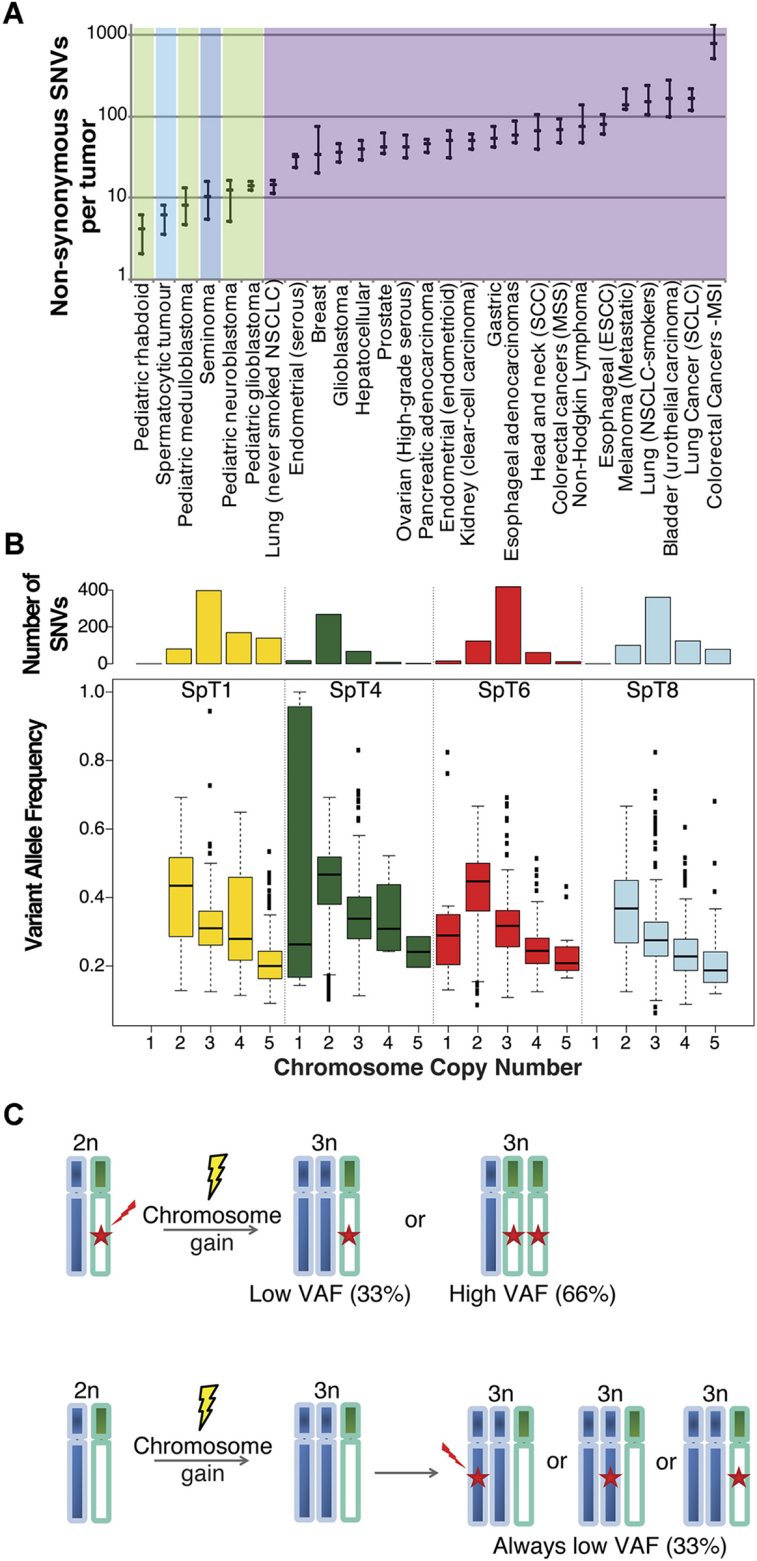
Single nucleotide variant analysis. **(A)** Mutation burdens in different tumor types. Compared to solid tumors with an adult age of onset (purple), SpT (light blue) have an extremely low number of non-synonymous SNV mutations, similar to pediatric cancers (green) and slightly lower than classical germ cell tumors (dark blue). Median values and interquartile range are presented. Data for other cancers from Vogelstein et al. 2013 [50]; Brabrand et al. 2015 [46]. **(B)** Box plots of the variant allele frequency observed in each SpT, binned by the copy number of the chromosome on which each SNV is located. Variant allelic ratios tended to be low (< 50%) and inversely correlated with the chromosome copy number on which they occurred (indicated at the bottom of the Figure). The total number of variants reported is shown as a bar plot at the top of the panel B. Allelic ratio data for non-synonymous SNVs are given in S3A Table; data for individual chromosomes are given for each tumor in S4 Table. (**C)** Schematic explaining the impact of mutational event order on expected variant allele frequency (VAF) in the case of (3n) chromosome number. In the upper panel, an SNV has occurred before the event of chromosome gain/duplication leading to trisomy. This will result in the observation of either high variant allele frequency (~66%) or low VAF (~33%) for the acquired SNV, depending which chromosome has been duplicated (green or blue, respectively). Assuming the SNV is a ‘passenger’ mutation, the two scenarios are anticipated to be observed with equal frequency; for a ‘driver’ mutation, the high VAF (66%) would be observed more commonly. In the lower panel, chromosomal gain/duplication has preceded the occurrence of the SNV. In this case, the VAF of the acquired SNV will always be observed at a low level (≤33%).

Given the extensive tumor aneuploidy associated with SpTs, somatic SNV identification of the four tumor-matched control pairs was performed using two different calling algorithms (Platypus [29] and MuTect2 [30]) in order to maximize the specificity of the variant calls. Across the four tumors, a total of 37 coding variants were called by both algorithms, including 24 non-synonymous exonic variants (Fig 2 and S3A Table); among this latter group, 22 (92%) variants were validated by dideoxy-sequencing (data not shown). A further 31 non-synonymous exonic calls made by a single algorithm (8 by MuTect2 and 23 by Platypus) were visualized in Integrative Genomics Viewer (IGV) [31] to rule out gross-alignment or mis-mapping errors. From this analysis, another variant in *NPHS1* present at 38% (22/58 mutant reads), was validated by dideoxy-sequencing. This variant was identified in SpT6 and was not called by MuTect probably because the sample N6T, the normal matched control to SpT6, exhibited 1/25 mutant read (S3A Table). This analysis shows that the strategy of using two calling algorithms provides high specificity without compromising on sensitivity (estimated > 90%) for somatic variant detection. Hence, we applied this approach genome-wide and overall an average of 610 somatic variants were identified by both algorithms across the four tumors (range: 362–786) (S3B–S3G Table). This number corresponds to an extremely low genome-wide mutational burden of ~0.2 SNV per Mb. The number of SNVs per tumor was highly correlated to the total chromosome number (r = 0.97; p-value = 0.025). No small indels and multiple nucleotide variations (MNV) located near or within exons were called by both algorithms, while indel calls made by a single algorithm (3 indels by MuTect2 and 19 indels by Platypus) were visualized in IGV and confirmed to be false positives. No single variant was shared across tumors and no known selfish or oncogenic mutations were identified (S3 Table). Overall, an average of 5.75 non-synonymous variants were identified per tumor, ranging from two variants (SpT4, near-diploid), five (SpT6), seven (SpT8) to nine (SpT1) per tumor, which indicates an overall genome-wide estimate of 0.2 (0.12–0.25) somatic SNVs/Mb (Fig 3A). Although we were not able to analyze somatically-acquired variants for the SpT3 singleton, coding variants not present in the reference genome were individually examined and compared to the COSMIC database confirming that no known driver mutation was present in the sample (data not shown).

Next, we examined the allelic ratio at which somatic variants were observed in the tumors. This analysis revealed that genome-wide the mutant allelic ratio tended to be low (< 50%) and inversely correlated with the chromosome copy number on which the mutations took place (Spearman rho = -0.42; p-value < 2.2 x 10^−16^) (Fig 3B). For the majority of variant calls these low mutant allelic ratios are consistent with the mutation being present only on a single chromosome (S4 Table). This observation implies that the chromosomal gains (n ≥ 3) must have preceded the acquisition of the somatic point mutations. Even in instances of UPD/LOH observed for three whole-chromosomes across two tumors, the mutant allele ratio was consistent with the somatic event having occurred secondarily on an already existing single copy of the parental chromosome—because 99% of the calls were observed at allelic ratio < 90% (S4 Table). This pattern of global low allelic frequency is best explained by a model in which the somatic SNVs identified in SpTs are late events in the pathogenesis of these tumors and hence are likely to represent passenger mutations (Fig 3C). The same pattern of low allelic ratio was also observed for the coding variants (S3A Table); hence, although some candidate pathogenic mutations were identified in the coding region of genes that have been associated previously with cancer, such as *MAP3K1* [32], *FAT1* [33], *POLR2A* [34], *LRP5* [35] and *PRPF8* [36], the low allelic ratio (median = 32.6%; range: 16–55%) at which the variants were observed in SpTs (S3A Table) is not mechanistically consistent with a pathogenic role for a driver mutation.

**Fig 4:**
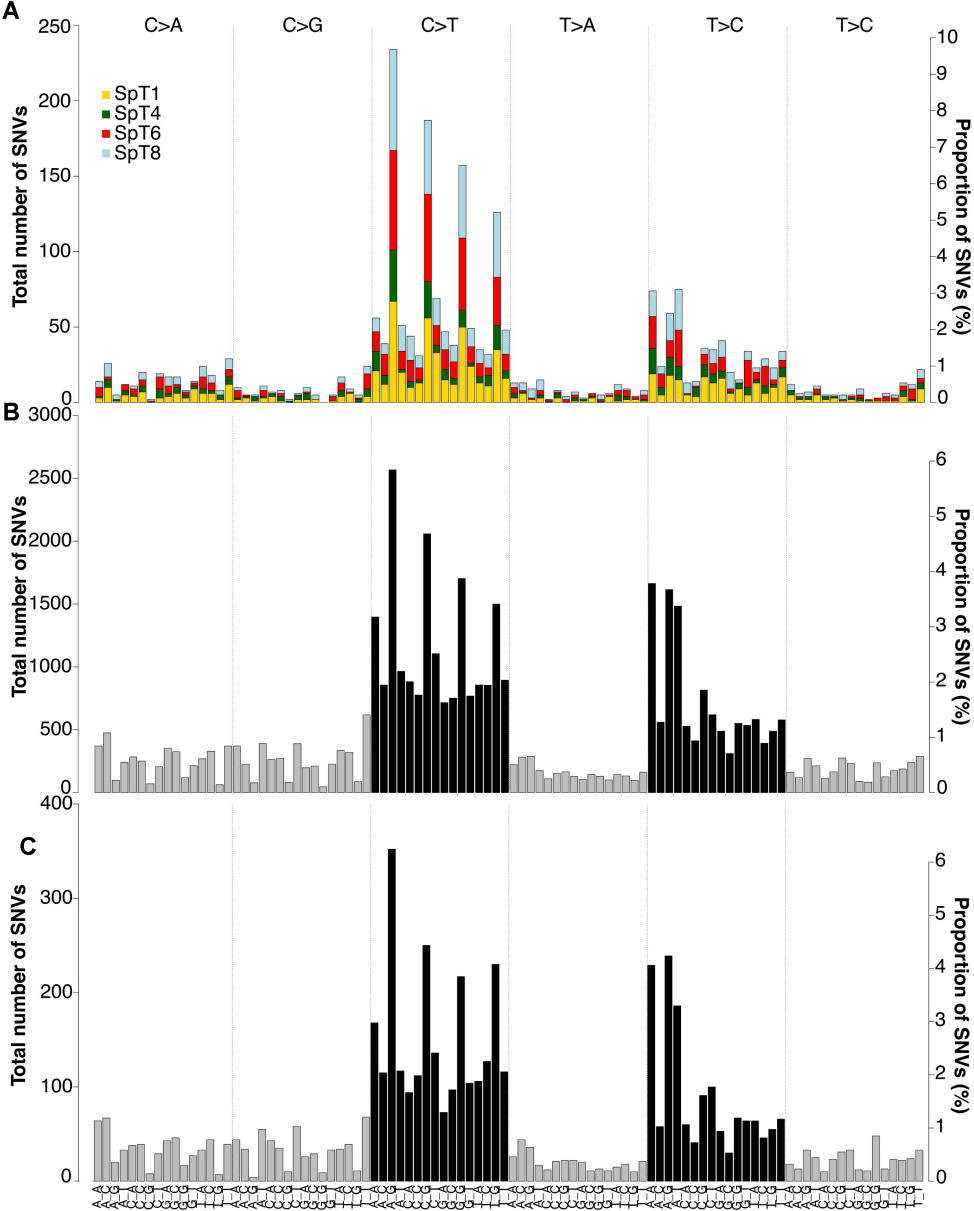
Trinucleotide analysis of somatic SNVs. **(A)** Trinucleotide context of somatic SNVs in SpT reveals a high frequency of mutations occurring at CpGs, most commonly in the ACG>ATG context. **(B)** The distribution of 43,942 *de novo* germline mutations identified from WGS of family trios reveals a strikingly similar profile. Data from Kong et al. 2012 [40], Goldmann et al. 2016 [43], Rahbari et al. 2016, [41] and Turner et al. 2016 [42]. **(C)** The profile of 5,640 paternally originating *de novo* germline mutations in also similar (Goldmann et al. 2016) [43]. In A-C, each of the six substitution subtypes: C>A, C>G, C>T, T>A, T>C, and T>G (top) are further divided by incorporating information on the bases immediately 5' and 3' (seen on the lower axis) generating 96 possible mutation types. The contribution made by each SpT is colour-coded in A; and in B-C, the black bars represent transitions, while grey bars are transversions.

Looking at the detail of the mutations identified genome-wide, all tumors showed a similar pattern of somatic substitutions: the majority (1821/2417; 75.3%) of the SNVs were transitions, with C>T (or G>A) transitions accounting for 51.4% (1243/2417) of all mutations. Among these, 56.6% involved CpG dinucleotides (704/1243; Fig 4A); transitions at CpGs represent the most common mutational signature in the human genome and are associated with a specific mutagenic mechanism involving deamination of 5-methylcytosine to thymidine. Spermatogonial stem cells display extensive and dynamic regulation of DNA methylation during development [37,38]. To assess whether methylation status in human testes may influence SpT mutation rate, we compared the locations of the SpT variants to methylation datasets for a testicular tissue sample and two well-characterized cell lines obtained by bisulfite-treated gDNA sequencing as part of the ENCODE Project Consortium [39]. Among the 1,151,596 CpG sites analyzed across the genome of a human testis from a 41-year old donor (GSM683850), 11 overlapped with sites that were mutated in SpTs. While genome-wide in the testicular tissue, 23.2% of CpG sites are associated with methylated regions (defined by more than 50% of methylated reads), in SpTs, we observed that 10 of the 11 overlapping transitions were in methylated regions, suggesting that in this tissue methylated CpGs are significantly more likely to mutate than unmethylated CpG sites (p-value = 3.87 x 10^−6^, binomial test). Association between SpT variants and methylation sites for the human embryonic stem cell line H1-hESC and the lymphoblastoid cell line (GM12878) was less significant (p-value = 1.31 x 10^−5^ and p-value = 0.037 respectively) (S5 Table).

Higher resolution SNV analysis, relying on the local trinucleotide context in which each mutation took place, showed a striking overlap (Pearson *r* = 0.92, p-value < 2.2 x 10^−16^) with the mutational profile documented for 43,942 high-confidence *de novo* germline mutations compiled from previously published WGS of 950 families [40,41,42,43] (Fig 4B) or a smaller SNV fraction that are known to have a paternal origin [43] (Fig 4C). We find that in SpTs, CpG transitions took place most frequently in the ACG>ATG trinucleotide context (Fig 4A); this represents a significant enrichment when compared to CCG>CTG, the second most frequent context (p-value = 0.0062, one-sided pair-wise *t*-test), a feature also observed for *de novo* germline mutations [41,43].

## Discussion

The integrated analysis of the mutational landscape of cancer genomes has provided a powerful approach to study specific mutational mechanisms leading to tumor formation but also affords a unique view, although through a ‘cracked lens’ [44], into the normal cellular processes shaping tissues of origin [45]. Although whole-exome sequencing (WES) was recently used to describe the mutational landscape of classical seminoma and non-seminoma, the most common forms of TGCTs [46,47,48,49], to our knowledge the present study represents the first WGS analysis of any type of TGCT. By providing a genome-wide overview of the mutational landscape acquired by tumor samples, WGS offers insights into the processes responsible for tumor pathology. Unlike classical TGCTs, which have an embryonic origin, SpTs are late adult-onset tumors that uniquely originate in the context of the post-natal germline [16,17] and therefore provide an opportunity to study the properties and mutational processes of a population of cells whose genetic integrity is crucial to the survival of our species.

Whilst somatic mutations in *FGFR3* and *HRAS* have previously been associated with SpTs [22,23], through the targeted screening of a panel of archival samples we identified two additional samples carrying a pathogenic mutation in *NRAS*; overall the mutation-positive tumours cluster significantly towards the older population of men with SpTs (Fig 1). By contrast, our investigation of the whole genomic landscape of five mutation-negative tumors (sampled from an average age range and to our knowledge the only collection of frozen samples) surprisingly indicates that genome-wide no candidate driver mutations could be identified in these samples. Instead, these TGCTs are characterized by a very ‘quiet’ and unusual mutational landscape that distinguishes them both from other TGCTs and other somatic adult-onset solid tumors. Analysis of somatic SNVs showed that the SpTs we sequenced carry an extremely low mutation load of ~0.2 SNVs/Mb, similar to, or lower than, pediatric tumors and about half of that estimated for classical TGCTs [50], despite the fetal origin of this latter tumor type (Fig 3A). This low mutation load is consistent with direct measurements of germline mutation rate based on parent-child trio WGS which concur that at ~1.2 x 10^−8^ per nucleotide per generation [40,43], the average human point mutation rate is several orders of magnitude lower than spontaneous mutation rates documented for somatic tissues [51]. Moreover, analysis of variant allelic ratio showed that genome-wide the allelic ratios at which acquired SNVs were observed are not consistent with a driver role and strongly suggest that these mutational events have occurred late in the tumor’s evolution and consist of randomly accumulating ‘passenger’ mutations (Fig 3B and 3C).

We observed that the majority of the 2,417 SNVs identified across the four SpTs were transitions (75.3%), with most of the C>T (or G>A) (56.6%) taking place at CpG dinucleotides, a signature characteristic of a mutational process known to be associated with methylation of CpG and involving their deamination to thymidine (TpG) [45]. Consistent with this observation, we showed an increased mutation load for transitions at CpGs in regions known to be methylated in adult testicular tissue. The SpT mutational signature is strikingly different from that found in the other TGCTs that originate during embryonic development and are characterized by relatively high C>A (or G>T) transversion and low C>T (or G>A) transition rates [46,48], a mutational pattern which is likely to reflect the global DNA demethylation reprogramming of primordial germ cells (PGCs) occurring during fetal development and maintained in the neoplastic precursor cells [52,53].

Higher resolution mutational spectra defined by the trinucleotide contexts in which SNVs take place showed that SpTs’ mutational signature is typical of germline *de novo* mutations (Fig 4) [41,43]. We observed a significant enrichment of transitions taking place in the specific sequence context ACG>ATG, which has also been documented for paternal germline *de novo* mutations (Fig 4C) [43]. Importantly, these findings suggest that most germline *de novo* mutations occur through the same mutational process and within a similar cellular environment as somatic mutations in SpTs. In addition, the low SNV mutation load observed in SpTs (and in agreement with the low trans-generational human mutation rate, measured through WES/WGS studies of family trios) highlights that the male germline is refractory to accumulation of *de novo* point mutations, pointing that DNA repair, apoptotic and/or cellular turnover mechanisms are likely to be under tight control in this tissue to curtail mutation rates [41,51].

By contrast to the low allelic ratios and mutation rates observed for SNVs, we report that the five SpTs we analyzed by WGS are characterized by non-random whole-chromosome aneuploidies. These findings are further supported by previously published data of another nine SpT cases [26,27] and considered together, relative gains of chr9 (14/14 cases) and chr20 (10/14 cases) as well as loss of chr7 (10/14 cases)—and to a lesser extent, chr13, chr15, chr22—are recurrently observed in SpTs (S2C Table). Notably, chr12 (or 12p), which is commonly gained in classical (type II) TGCTs (seminoma and non-seminoma) (54), was not altered over the basic ploidy number in any of the tumors documented so far (S2C Table). Moreover, chr9 and chr20 are not recurrently affected in classical TGCTs [47,48,49,54], further highlighting the distinct molecular pathology of SpTs.

Based on the observation that the aneuploidy pattern within each tumor is non-random (S2C Table) and appears to be stable over time, we propose that the initiating event driving oncogenesis in SpTs involves the whole-chromosome imbalance itself. A similar pathogenic mechanism has been proposed for other tumors characterised by similar properties such as high hyperdiploid childhood acute lymphoblastic leukemia. In these cancers, aneuploidies, through specific altered gene dosage, induce changes in gene expression profiles that cause proliferation and are responsible for promoting tumor phenotypes [55].

**Fig 5:**
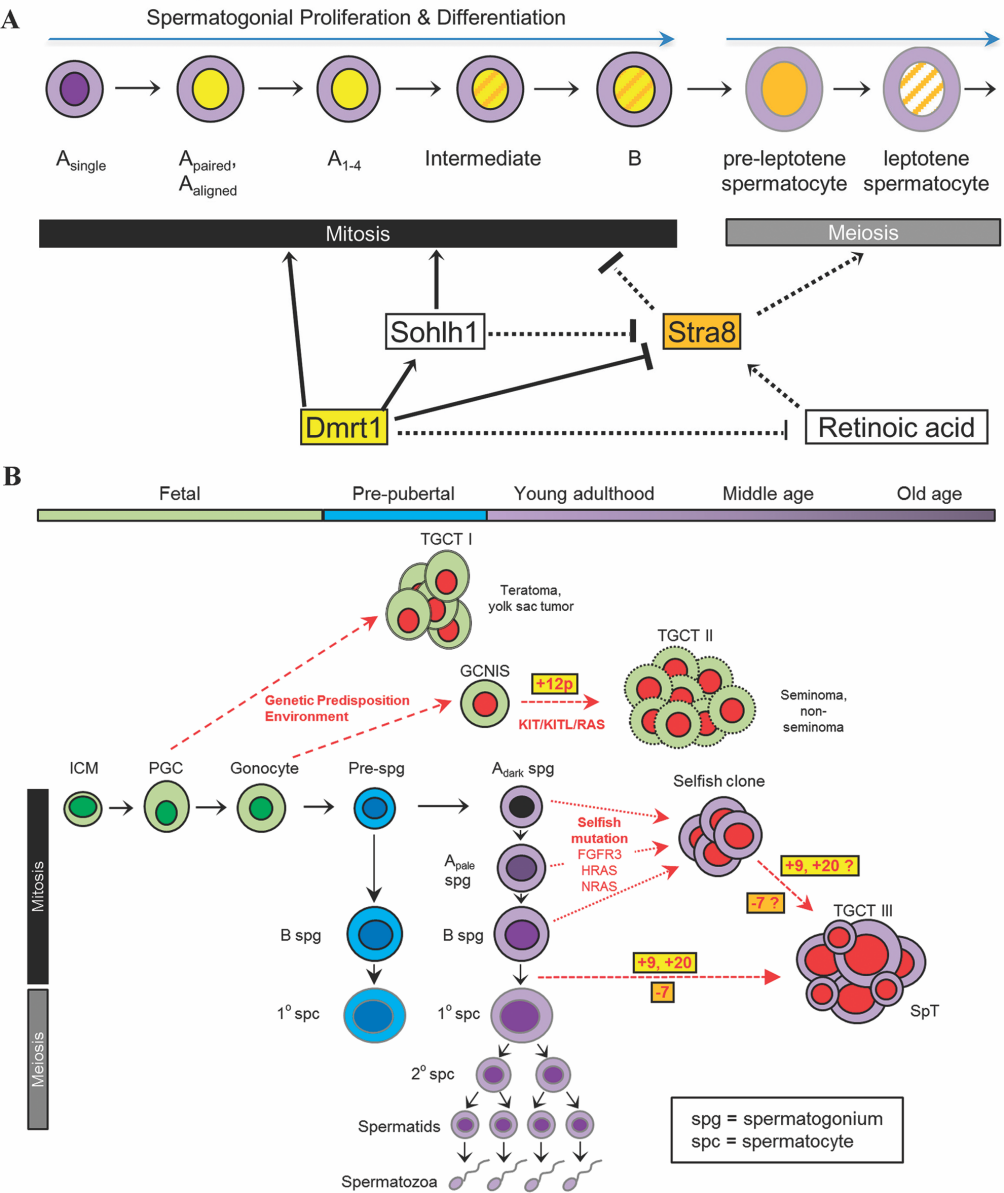
Model of SpT pathogenesis. **(A)** Model for the role of key regulators of the retinoic acid pathway in mitotic-meiotic transition. In murine spermatogonia, Dmrt1 inhibits meiosis via direct transcriptional repression of *Stra8*, indirect repression of the RA pathway, and the direct promotion of *Sohlh1* expression. *Dmrt1* expression is lost as B spermatogonia undergo the transition to pre-leptotene spermatocytes, resulting in upregulation of *Stra8* and subsequent meiotic progression. Gain of human chr9 (containing *DMRT1* and *SOHLH1*) and loss of chr7 (containing *STRA8*) in SpTs may drive expansion of tumor cells by altering the mitosis-meiosis transition leading to re-entry into mitosis. Model based on Matson et al. (2010). **(B)** Cellular development and differentiation during normal spermatogenesis and in TGCTs. In this schematic, age-related development proceeds towards the right and differentiation proceeds downwards. Impaired differentiation of primordial germ cells lead to type I TGCTs in infants. Differentiation arrest of gonocytes leads to germ cell neoplasia in situ [74], precursor cells that will develop into TGCT II in young adults. In early childhood, from mini-puberty, pre-spermatogonia (Pre-spg) begin to divide, mature into B-type spermatogonia and occasionally differentiate into primary spermatocytes (pathway coloured blue). Complete meiotic division and spermiogenesis begins at puberty. Post-pubertal spermatogonia (A_dark_, A_pale_ and B) proliferate and differentiate to form primary spermatocytes undergoing meiosis to form haploid spermatids which mature into spermatozoa (pathway coloured purple). SpTs (TGCT III) are proposed to be initiated post-natally (either during infancy or early puberty (blue) or during adulthood (purple)). During adulthood, selfish mutations in the RTK/RAS/MAPK pathway that arise spontaneously in adult spermatogonia confer growth/survival advantage to the mutant cells leading to clonal expansion over time, a universal process that occurs in the testes of all men as they age [20]. As SpT is extremely rare, the transition from selfish clone to SpT likely requires secondary mutagenic events such as whole-chromosome aneuploidy. Moreover, we speculate that infancy and/or early puberty may also constitute a period of susceptibility to the development of SpTs, through a block in the mitosis-meoisis transition caused by specific whole chromosome imbalance.

Without recurrent focal events and/or point mutations, it is difficult to delineate the minimal genomic regions that promote SpT pathogenesis. However, the presence of important genes on chromosomal regions subject to recurrent copy number imbalance enable us to develop a model of SpT pathogenesis (Fig 5A) that, although currently speculative, can be used as a framework and tested by future experiments and observations. We note, first, that human chr9 and chr7 carry genes that are known to be dosage-sensitive regulators of the mitosis-meiosis transition (Fig 5A). Interestingly, in the two tumors with gains of chr9 arm tips, the regions gained encompass the *DMRT1* (Doublesex And Mab-3 Related Transcription Factor 1) gene located on 9p24.3 and *SOHLH1* (Spermatogenesis and oogenesis specific basic helix-loop-helix 1) locus on 9q34.3 (in SpT4 and SpT6, respectively) (Fig 2, S3 Fig). In mouse, *Dmrt1* has a crucial role in coordinating the mitosis-meiosis progression via a dual mechanism; (1) it promotes spermatogonial development by activating spermatogonial differentiation genes, such as its direct target *Sohlh1* and (2) it supports self-renewal by repressing the entry into meiosis of undifferentiated spermatogonia via inhibition of retinoic acid (RA)-response genes such as *Stra8* (stimulated by retinoic acid 8), a gene required for initiation of the meiotic program and spermatogonial differentiation in mouse testes [56,57]. *Stra8*-deficient mouse testes lack meiotic and post-meiotic cells and accumulate undifferentiated type A spermatogonial cells that progressively invade the seminiferous tubules, causing gross overgrowth in ~50% of testes in aged mice [58]. This phenotype is reminiscent of intratubular SpT, a lesion believed to be the precursor to SpT that has been observed in some instances alongside SpTs [1,17]. Intriguingly, *STRA8* is located on human chr7, copies of which are lost in 10/14 of SpT cases, suggesting that the expansion of tumor cells may be driven by an altered balance of RA pathway effectors that converge to inhibit the mitosis-meiosis transition.

DMRT1 has also been proposed to be a key negative regulator of meiotic entry in the human gonads. DMRT1 protein is strongly expressed in spermatogonia type-A (A-pale) and type-B, but is not detectable in leptotene spermatocytes, suggesting that down-regulation of this factor is crucial to the progression of meiosis [19]. Moreover, *DMRT1* has previously been shown to be over-expressed in SpTs [27,59]. Other factors outside the RA pathway that may be relevant to the pathology of SpTs include the *Xeroderma Pigmentosum Type A (XPA)* gene located on chr9q22.3, a key regulator of the NER (Nucleotide-Excision Repair) pathway that is a diagnostic marker for SpT [9]; the DNA Methyltransferase *DNMT3b* and the cancer testis antigen (CTA) *BORIS* ((brother of the regulator of imprinted sites, also known as CTCFL (CCCTC-binding factor-like)); both genes are known to be up-regulated in SpTs [27] and are located on chr20, which is gained in 11/14 SpTs (S2C Table). Moreover, *Boris* mutant mouse testes are smaller than their wild-type counterparts because spermatogonia fail to enter meiosis and undergo apoptosis [60].

As outlined in Fig 5A and 5B, we speculate that the combination of simultaneously acquired gains of chr9/chr20 (leading to increased expression of molecules promoting mitosis such as DMRT1, SOHLH1, BORIS) and loss of chr7 (associated with reduced expression of the meiotic promoting factor STRA8) converge to alter the tight genetic circuitry responsible for controlling the mitosis-meiosis transition; this rare combination of factors could provide a unique stage in which differentiating spermatogonia unable to proceed through meiosis (because of low STRA8 levels), are instead instructed to re-enter a proliferative course. Consistent with the characteristic presence of three different cell types observed in SpTs and cytometric analyses of DNA content [14,15], this process is likely to be relatively ‘leaky’, occasionally allowing cells to enter the differentiation pathway and initiate meiosis. Of note, SpTs are associated with a high apoptotic index that may be indicative of the failure to complete the mitosis-meiosis transition [61]. This proposal is further supported by the simultaneous expression of meiosis regulators (DMRT1, SCP3, CYPB26B1, XPA) and spermatogonial markers (MAGEA4, FGFR3, SSX, SAGE1) in most SpTs [9,17,18,59].

In this scenario, it is unclear at which stage of development the SpTs would arrest before re-entering mitotic proliferation. In budding yeast, commitment to meiosis (and sporulation) does not occur until the end of meiotic prophase I, a stage at which DNA has already been replicated, homologs have paired and recombination has been initiated. Up to this point, if culture conditions are modified, cells may re-enter mitosis and ‘return to growth’, a process precisely controlled by CDK and cyclin genes and that allows cells to faithfully segregate whole chromosomes; dysregulation of this process in mutant yeast cells leads to an aberrant division pattern, an increase in genome copy number and chromosomal aneuploidy [62]. In the nematode *C*. *elegans* hermaphrodite germline, the mitosis-meiosis transition can also be reversed through a process called dedifferentiation. In this system, the key mediators of the mitosis-meiosis transition are PUF (Pumilio and FBF) RNA-binding proteins, and removal of the PUF-8 protein leads to formation of germline tumors that derived from primary (and occasionally secondary) spermatocytes. Interestingly, the mild phenotype of the PUF-8 mutants is greatly enhanced by the activation of the MAPK pathway [63], probably by promoting both dedifferentiation and proliferation. We envisage that a similar process, although relying on the concerted activation of different mitosis-meiosis regulators present on specific chromosomes, may also be operating in the mammalian testis.

Our targeted screening approach in the present and previous studies has shown that a subset of SpTs carries well-known driver mutations in *FGFR3* [22], *HRAS* [22,23] and *NRAS* (Fig 1A). Strikingly, all mutation-positive samples were diagnosed at ≥ 55 years. However, it is unlikely that these oncogenic mutations are sufficient to cause SpTs on their own. Indeed, it has been shown that these and similar mutations are associated with benign intratubular ‘selfish’ clonal expansions of spermatogonia that progressively accumulate in the testes of all men as they age [20,24], while SpTs are very rare occurrences. Moreover, we have shown that most selfish clones caused by strongly activating mutations are characterized by impaired spermatogenesis and the absence of differentiating haploid gametes [24]; hence this developmental block may constitute an early predisposing event in SpT tumorigenesis. Of the 11 SpTs in which *FGFR3/HRAS/NRAS* mutations have been identified so far, chromosomal copy number information is available for only one tumor (SS2) from an 84 year old man [23]. Interestingly, similar to the five cases we studied by WGS, this tumor has been previously shown to exhibit gains of chr9 and chr20 [27] (S2C Table), suggesting that a molecular mechanism via a specific combination of the same non-random chromosomal aneuploidies may be responsible for the rare transition from common benign intratubular spermatogonial clone to SpT (Fig 5B). Unfortunately, because of the lack of good quality samples, we have not been able to assess the chromosomal copy numbers of the other mutation-positive samples to test this hypothesis.

In summary, our findings highlight that SpT biology reflects the unique properties of the male germline. Because of evolutionary constraints to maintain genomic integrity across generations, the male germline is refractory to the accumulation of *de novo* mutations. Nevertheless, SpTs may be exploiting a unique feature of the male germline, its ability to undergo both mitosis and meiosis. We speculate that SpTs occur rarely because the oncogenic driver events are caused by rare catastrophic instability involving specific combinations of whole-chromosome gains and losses. We propose that this oncogenic mechanism of altered gene expression via whole-chromosome aneuploidies represents a rare vulnerability of the post-natal male germline, which may be intimately linked to the failure to complete the process of mitosis-meiosis transition.

## Material and methods

### Samples

For WGS, tumor (SpT1, SpT3, SpT4, SpT6, SpT8) and matched normal (N1B, N4B, N6T, N8T) samples were collected in the Netherlands and stored in liquid nitrogen prior to DNA extraction. The use of tissue samples remaining after diagnosis for scientific reasons was approved by the Medical Ethical Committee of the Erasmus MC Rotterdam (The Netherlands), (MEC 02.981). This included the permission to use the secondary tissue without further consent. Samples were used according to the “Code for Proper Secondary Use of Human Tissue in The Netherlands” developed by the Dutch Federation of Medical Scientific Societies (FMWV (Version 2002, update 2011)). Tumors SpT1, SpT3 and SpT4 have been reported before [15,27] and the diagnosis of SpT was performed by an experienced pathologist and supported by immunohistochemistry. Of note, the 48 year old patient diagnosed with SpT4, had another tumor (H6T (S1 Table)) in the contralateral testis (i.e. bilateral case) that was not WGS sequenced; based on dideoxy-sequencing, none of the non-synonymous variants identified in SpT4 were present in the H6T contralateral tumour. DNA was extracted from frozen tumor tissue at the same time as the matched control samples consisting of either blood (N1B; N4B) or pathologically normal testicular tissue adjacent to the tumor (N6T; N8T). The majority of the formalin-fixed paraffin embedded (FFPE) tumor samples have been described previously (S1 Table), and the six new samples were collected in the Netherlands (SS_46, SS_49, SS_50, SS_51 and SS_53) or Denmark (SS_14) and were processed following the same standard protocol [22,23].

### Whole-genome sequencing and quality control

Whole-Genome sequencing was performed as part of the WGS500 consortium. Sequencing library preparation and Illumina sequencing, quality control and read mapping strategies are described in [64]. Sequencing was performed on the Illumina HiSeq 2000 by the Oxford Genomics Centre at the Wellcome Trust Centre for Human Genetics. We generated 100-bp reads using v2.5 sequencing chemistry with a minimum of 1.5 billion reads (52x) for the tumors and 796 million reads (26x) for the matched control samples. Quality control of the sequencing data was performed using FastQC [65] and read mapping was performed using Stampy v1.0.12–1.0.13 [66].

### Variant calling and quality control

Acquired single nucleotide variants (SNVs), small insertions/deletions (indels) and multiple nucleotide variants (MNVs) were detected using two algorithms. Platypus v0.8.1 [29] uses a local realignment and assembly algorithm to accurately identify SNVs and short indels. We first identified mutations by jointly calling each tumor sample with its matched normal sample. The resulting set of variants was further processed using a likelihood model that computes a posterior probability for each somatic variant (scripts provided on the Platypus GitHub repository https://github.com/andyrimmer/Platypus/blob/master/scripts/findSomaticMutationsInTumour.py). Variants with a posterior probability > 1 (Phred-scale) were retained. We also performed variant calling using MuTect2 v1.1.6 [30], a somatic SNP and indel caller that is part of Genome Analysis Toolkit (GATK) 3.5–0. Mutect2 relies on a Bayesian classifier method to detect somatic mutations with very low allele fractions and utilizes tuned filters to ensure high specificity. We used the default settings of the algorithm and retained the variants flagged as “PASS”. Variants that were identified by both algorithms were prioritized for further analysis. Variants were annotated using ANNOVAR [67] with respect to RefSeq genes.

### Germline variants

Germline variants present in matched normal samples were called using Platypus v0.8.1 [29] and annotated using ANNOVAR [67]. We identified 1330 non-synonymous SNVs and indels that were common across all samples. Out of these only 2 SNVs and 1 indel had a population frequency < 1% based on 1000 Genomes populations and the Exome Aggregation Consortium (ExAC) release (v0.3) databases. Manual inspection of these three calls on IGV (Integrative Genomics Viewer) showed that they were false positives.

### Detection of structural variation, chromosome copy number and ploidy levels

We used Samtools [68] to extract the read depth in base positions that correspond to one million SNP markers typed by the Illumina Human 1M array. A ratio between the tumor and normal read depth was calculated at every SNP position. A moving average of read depth was calculated using a window of 500 SNPs. Using the read counts of every base in each SNP position, we calculated the B allele frequency (BAF) defined as the proportion of allele-specific read counts of each SNP. Manual inspection of both read depth ratio and BAF was initially used to infer the whole-chromosome alterations. These large-scale copy number alterations and loss of heterozygosity were confirmed using OncoSNP-SEQ [28], a statistical model-based approach for inferring copy number profiles directly from high-coverage whole-genome sequencing data. To reduce false positives, only OncoSNP-SEQ calls obtained for more than 1500 SNP-probes were considered. Using this algorithm, the tumor purity (contamination by normal cells) and ploidy level (average number of reads for a unit copy number change—i.e., the haploid coverage) could be inferred for each tumor. Contamination was shown to be minimal for most tumors, except for SpT8 that exhibited an estimated 15–20% wild-type contamination. Furthermore, to study structural variation at a gene-by-gene level, for each tumor sample and its paired normal control, we extracted the average read coverage for each exon of every RefSeq gene in the human genome (hg19). We calculated the ratio of tumor vs. normal read depth for each exon and extracted all exons showing deviation from the expected ratio, that is, corresponding to more than one unit haploid copy number change. The software FACTERA [69] with the default settings was used to detect gene fusions and structural variants including deletion, duplication, inversion and translocation. Circos plots that included tracks showing the read depth and BAF were created using modified functions from the R package RCircos [70].

### Statistical analysis of data

A permutation test was used to assess the significance of recurrent chromosome gains (or losses). We performed 100,000 permutations of autosome copy numbers and calculated the empirical p-values by counting the number of times the sum of copy numbers for each chromosome exceeded (or was below) that of the observed sum. One copy number per chromosome was used for this analysis; in the case of chromosomes with specific arm amplifications, only the copy number of the whole-chromosomes was used. The P-values were adjusted for multiple testing using the Benjamini-Hochberg correction method. To characterize the variant allele frequencies (VAFs) for each tumor within each chromosome region, we calculated the 50^th^ (median), 90^th^ and 99^th^ percentiles of the VAF distribution.

### SpT mutational spectrum and signatures of germline *de novo* mutations

To derive the mutational spectra of SNVs, we classified all mutations based on the reference and mutant alleles found at each SNV site and further stratified them based on their tri-nucleotide context. The SNVs were initially classified based on the following substitutions: C:G>A:T, C:G>G:C, C:G>T:A, T:A>A:T, T:A>C:G, and T:A>G:C. These were further refined by including the sequence context of each mutated base (5’ and 3’ of the mutated base), resulting in 96 mutation types. We created a high-confidence set of germline *de novo* mutations (DNMs) from four studies [40,41,42,43]. For the dataset from [42], we only included DNMs called by both callers as defined in the study. Furthermore in the lower panel of Fig 3C, we included all paternally-originating DNMs phased in [43]. The mutational spectra were derived for all DNMs as described above.

### Methylation data

Reduced-representation bisulfite sequencing (RRBS) methylation data were downloaded from the UCSC server (ENCODE [39] for three samples: BC_Testis_N30 (testis of a 41-year-old Asian donor), GM12878 (B lymphocytes cell line from a European Caucasian donor) and H1-hESC (embryonic stem cell line). Only sites common to the replicate datasets were included in the analysis, consisting of a total of 1,151,596 sites for BC_Testis_N30, 1,048,775 sites for GM12878 and 1,118,911 sites in H1-hESC. For each ENCODE sample, sites for which more than 50% of the reads were methylated in both replicates were considered to be methylated and those below this threshold, to be unmethylated. Locations of the ENCODE sites were compared with the genomic positions of the SpT variant calls. We computed binomial P-values as Bin(q, n, p), where q is the number of methylated SpT variants, n is the total number of SpT calls for which methylation data were available and p is the proportion of sites that were methylated in the ENCODE data set.

### SNV validation and SpT resequencing

For validation by dideoxy-sequencing, we used the Primer3 software [71] to design primers specific for the region to amplify by PCR; each primer was tailed with a common sequence (CS1 or CS2) that was used for sequencing (S6B Table). For SpT resequencing, 68 single molecule molecular inversion probes (MIPs) were designed to target 145 selfish mutation hotspots in *FGFR2*, *FGFR3*, *HRAS*, *KRAS*, *NRAS*, *PTPN11* and *RET* using the MIPGEN algorithm [72] (S6A Table). The MIP protocol is as in described in [73] with some minor modifications. After an initial assessment of the capture yield of each MIP, the probes were divided into pools of 44 (Pool 1 –high performer) and 24 (Pool 2—low performer) and phosphorylated using T4 Polynucleotide Kinase (NEB) (0.4 U per μl of 100μM MIPs) at 37°C for 45 min, followed by heat inactivation at 65°C for 20 min. 200 ng of sample gDNA was incubated with each MIP pool, at a 4000:1 molar ratio of MIPs:DNA, and samples were denatured for 10 min at 95°C, followed by 24 hr incubation at 60°C with 3.2 U polymerase (Hemo Klentaq (NEB)) and 1U ligase (Ampligase (Epicentre)). Template DNA and unbound MIPs were removed by incubating with 1 U exonuclease I (NEB) and 5 U exonuclease III (NEB) for 45 min, followed by heat inactivation at 95°C for 2 min. Circularized MIPs with captured regions were amplified and barcoded by PCR using primers targeting consensus sequences on the MIP backbone (S6B Table). Barcoded products from Pools 1 and 2 were combined, gel extracted and sequenced on Ion PGM 314 or 316 chips (Life Technologies). Variants at the 145 mutational hotspots with a minimum frequency > 0.1 and minimum coverage of 20x were called using Ion Torrent variantCaller (v4.2.1.0). Manual inspection was also performed for 45 hotspots, where overlapping reads from the ligation or extension arms of MIPs targeting the alternative strand may have affected the apparent variant frequency. Two regions in *NRAS* (p.G12/13 and p.Q61) and one in *KRAS* (p.G12/G13) and in *FGFR3* (p.A265-p.Y278) were poorly covered in some samples and were subsequently amplified by PCR and dideoxy-sequenced. Overall, 80.2% (range 38.1%–91.2%) of target codons were covered in all samples (S6C Table). Variants in WGS samples and MIP-screened samples were validated by PCR amplification and dideoxy-sequencing (S6B Table).

## Supporting information

S1 Fig Identification of an NRAS c.182A>G (p.Q61R) mutation in two spermatocytic tumor samples. **(A)** Heterozygous *NRAS* c.182A>G (p.Q61R) mutations in samples SS8 (age 86) and H8T-1 (age 55) identified in the MIP screen, visualised in IGV. **(B)** Variant validation by PCR amplification and dideoxy-sequencing. The *NRAS* c.182A>G mutations were validated in both SS8 and H8T-1. The mutation was also present in an additional biopsy (H8T-2) from the same tumour as H8T1. The red boxes represent the frame of the codon affected and arrows indicate the presence of a mutant ‘G’ peak. The mutation was not detected in control (ctrl) DNA.
(PDF)

S2 Fig Circos plot of SpT3. The first (outer) circle represents chromosomal copy numbers (inferred from relative sequencing depth); color indicates chromosomal copy numbers as described in key; the second ring shows the relative allele frequency of the minor (B-allele) for one million common SNPs; the third ring indicates chromosome number and locations. The tumor name and ploidy number are indicated in the middle.
(PDF)

S3 Fig Gain of chr9 arms in SpT4 and SpT6. **(A)** Relative sequencing read coverage depth of SpT4 to matched normal sample N4B. SpT4 is near-diploid (see Fig 2), but chr9 is mainly present in 3 copies, with regions of the tip of chr9p present at 4 and 5 copies. This sub-amplified region of chr9p tip contains *DMRT1*, a key regulator of mitosis-meiosis transition (breakpoint locations are given in S2 Table). **(B)** Relative sequencing read coverage depth of SpT6 to matched normal sample N6T. SpT6 is near- triploid (see Fig 2), but chr9 is mainly present at 4 copies, with the tip of chr9q present at 5 copies. This sub-amplified region contains the known *SOHLH1*, a regulator of spermatogonial differentiation (breakpoint locations are given in S2 Table).
(PDF)

S1 Table List of SpT samples analysed in the present study, including multiple identifiers (ID) used in previous studies and targeted sequencing results. (PDF)

S2 Table Chromosome Copy number variation and breakpoints of SpT samples. (XLSX)

S3 Table SNVs and indels calls. (XLSX)

S4 Table Percentiles of Variant Allele frequencies (VAFs) of WGS spermatocytic seminoma samples binned by chromosomal regions estimated by OncoSNP-SEQ. (XLSX)

S5 Table Summary of the ENCODE methylation dataset for 3 tissue samples and comparison with SpT mutations. (PDF)

S6 Table Molecular inversion probe design, amplification primers, and target coverage analysis. (XLSX)

## Abbreviations

SpT: Spermatocytic Tumor
SSC: Spermatogonial Stem cell
PGC: Primordial Germ Cell
TGCT: Testicular Germ Cell Tumor
SNV: Single Nucleotide Variant
BAF: B-Allele Frequency
GCNIS: Germ Cell Neoplasia In Situ
LOH: Loss of Heterozygosity
