# Supplementary material for: Whole-genome sequencing of spermatocytic tumors provides insights into the mutational processes operating in the male germline
Source: PLoS One. 2017 May 22;12(5):e0178169. doi: 10.1371/journal.pone.0178169 (PMC5439955; doi:10.1371/journal.pone.0178169)
Supplement: S1 Fig — (A) Heterozygous NRAS c.182A>G (p.Q61R) mutations in samples SS8 (age 86) and H8T-1 (age 55) identified in the MIP screen, visualised in IGV. (B) Variant validation by PCR amplification and dideoxy-sequencing. The NRAS c.182A>G mutations were validated in both SS8 and H8T-1. The mutation was also present in an additional biopsy (H8T-2) from the same tumour as H8T1. The red boxes represent the frame of the codon affected and arrows indicate the presence of a mutant ‘G’ peak. The mutation was not detected in control (ctrl) DNA. (PDF) [file pone.0178169.s001.pdf]

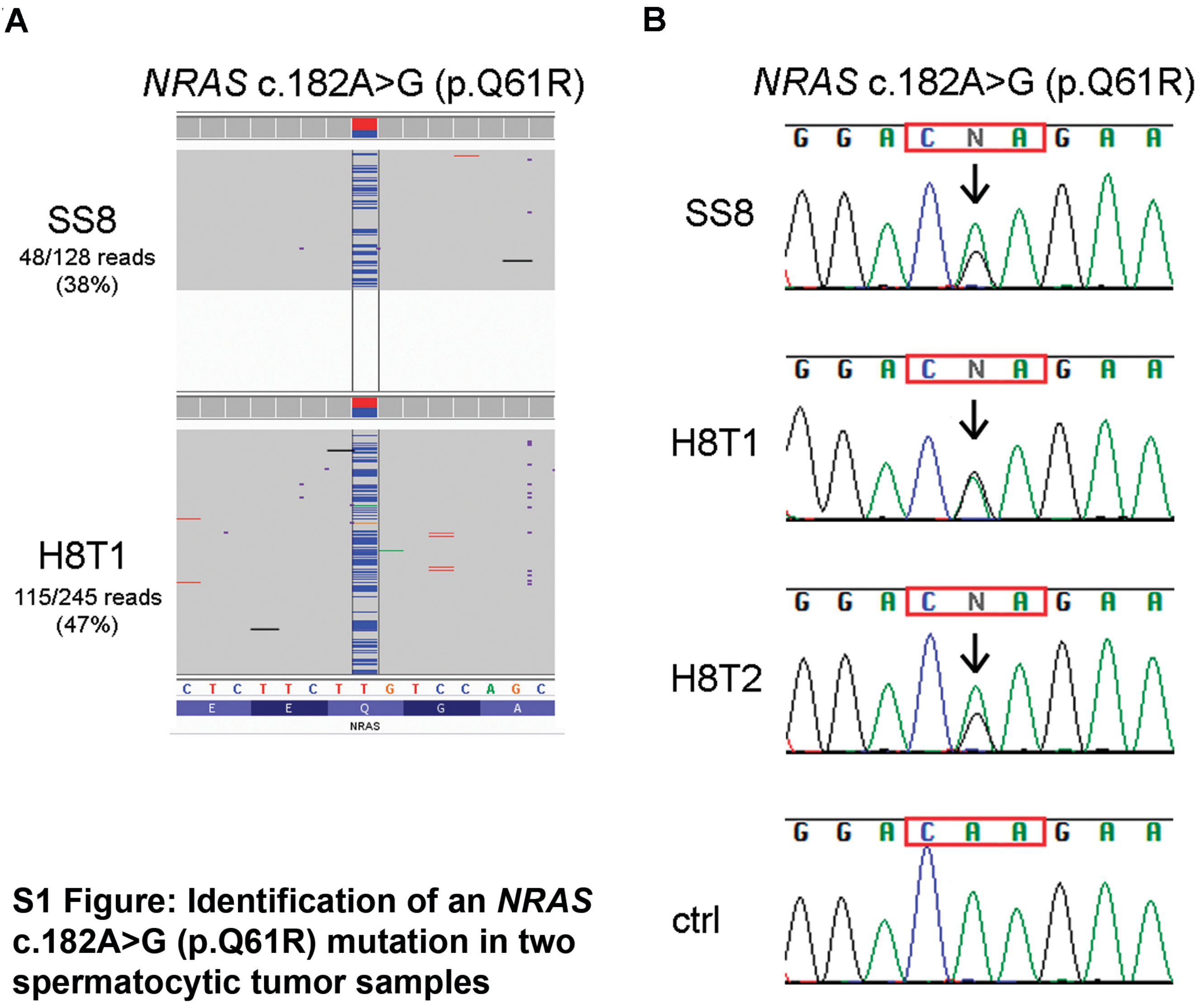

S1 Figure: Identification of an *NRAS* c.182A>G (p.Q61R) mutation in two spermatocytic tumor samples

Identification of an NRAS c.182A>G (p.Q61R) mutation in two spermatocytic tumor samples. (A) Heterozygous NRAS c.182A>G (p.Q61R) mutations in samples SS8 (age 86) and H8T-1 (age 55) identified in the MIP screen, visualised in IGV. (B) Variant validation by PCR amplification and dideoxy-sequencing. The NRAS c.182A>G mutations were validated in both SS8 and H8T-1. The mutation was also present in an additional biopsy (H8T-2) from the same tumour as H8T1. The red boxes represent the frame of the codon affected and arrows indicate the presence of a mutant ‘G’ peak. The mutation was not detected in control (ctrl) DNA.
