# Supplementary material for: Whole-genome sequencing of spermatocytic tumors provides insights into the mutational processes operating in the male germline
Source: PLoS One. 2017 May 22;12(5):e0178169. doi: 10.1371/journal.pone.0178169 (PMC5439955; doi:10.1371/journal.pone.0178169)
Supplement: S2 Fig — The first (outer) circle represents chromosomal copy numbers (inferred from relative sequencing depth); color indicates chromosomal copy numbers as described in key; the second ring shows the relative allele frequency of the minor (B-allele) for one million common SNPs; the third ring indicates chromosome number and locations. The tumor name and ploidy number are indicated in the middle. (PDF) [file pone.0178169.s002.pdf]

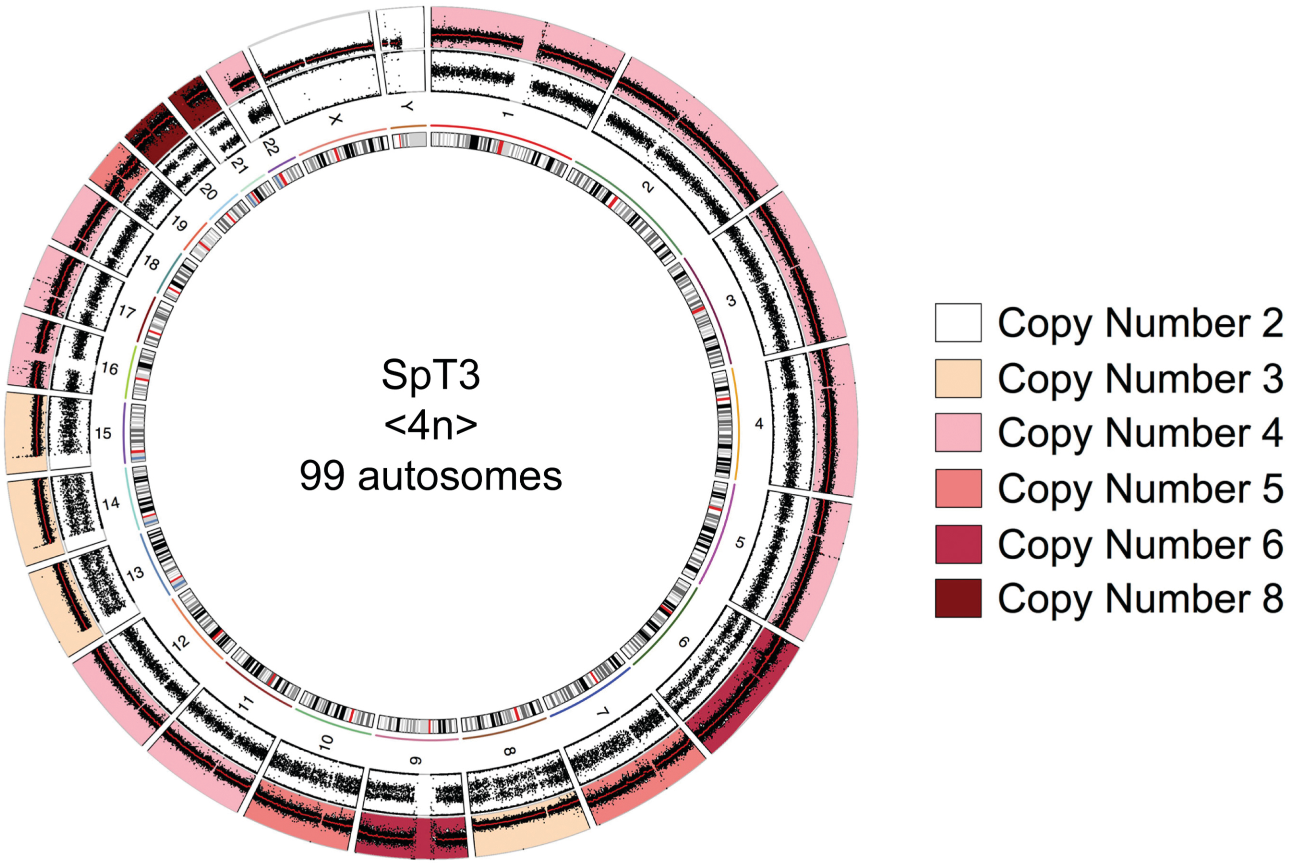

S2 Figure: Circos plot of SpT3

The first (outer) circle represents chromosomal copy numbers (inferred from relative sequencing depth); color indicates chromosomal copy numbers as described in key; the second ring shows the relative allele frequency of the minor (B-allele) for one million common SNPs; the third ring indicates chromosome number and locations. The tumor name and ploidy number are indicated in the middle.
