# Supplementary material for: Whole-genome sequencing of spermatocytic tumors provides insights into the mutational processes operating in the male germline
Source: PLoS One. 2017 May 22;12(5):e0178169. doi: 10.1371/journal.pone.0178169 (PMC5439955; doi:10.1371/journal.pone.0178169)
Supplement: S3 Fig — (A) Relative sequencing read coverage depth of SpT4 to matched normal sample N4B. SpT4 is near-diploid (see Fig 2), but chr9 is mainly present in 3 copies, with regions of the tip of chr9p present at 4 and 5 copies. This sub-amplified region of chr9p tip contains DMRT1, a key regulator of mitosis-meiosis transition (breakpoint locations are given in S2 Table). (B) Relative sequencing read coverage depth of SpT6 to matched normal sample N6T. SpT6 is near- triploid (see Fig 2), but chr9 is mainly present at 4 copies, with the tip of chr9q present at 5 copies. This sub-amplified region contains the known SOHLH1, a regulator of spermatogonial differentiation (breakpoint locations are given in S2 Table). (PDF) [file pone.0178169.s003.pdf]

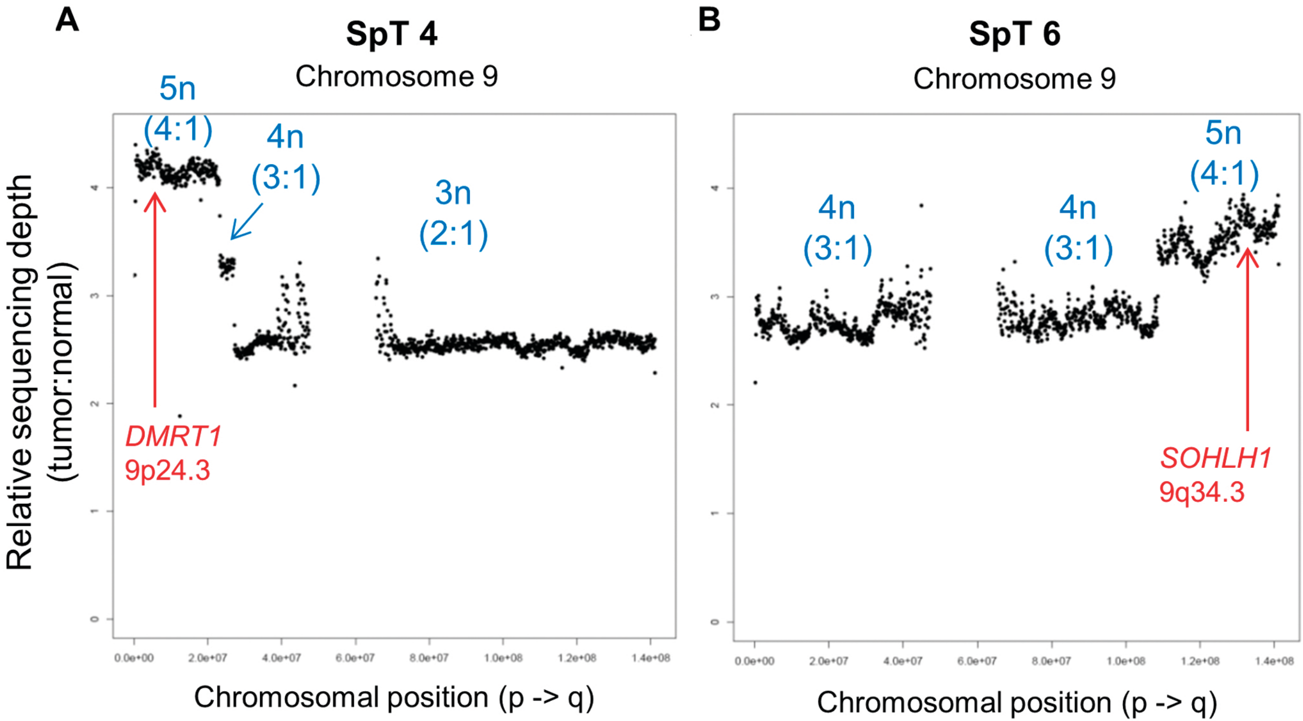

S3 Figure: Gain of chr9 arms in SpT4 and SpT6

(A) Relative sequencing read coverage depth of SpT4 to matched normal sample N4B. SpT4 is near-diploid (see Figure 2), but 3 integral copies of chr9 are present, with regions of the tip of chr9p present at 4 and 5 copies. This sub-amplified region of chr9p tip contains DMRT1, a key regulator of mitosis-meiosis transition (breakpoint locations are given in S2 Table).

(B) Relative sequencing read coverage depth of SpT6 to matched normal sample N6T. SpT6 is near- triploid (see Figure 2), but 4 integral copies of chr9 are present, with the tip of chr9q present at 5 copies. This sub-amplified region contains the known SOHLH1, a regulator of spermatogonial differentiation (breakpoint locations are given in S2 Table).
