# Supplementary material for: Whole-genome sequencing of spermatocytic tumors provides insights into the mutational processes operating in the male germline
Source: PLoS One. 2017 May 22;12(5):e0178169. doi: 10.1371/journal.pone.0178169 (PMC5439955; doi:10.1371/journal.pone.0178169)
Supplement: S1 Table — (PDF) [file pone.0178169.s004.pdf]

S1 Table. List of SpT samples analysed in the present study, including multiple identifiers (ID) used in previous studies and targeted sequencing results

ID used in the present study[(1)]	Looijenga et al. 2006	Goriely et al. 2009 [(2)]	Giannoulatou et al. 2013 [(3)]	Material source (FFPE or Frozen)	Age at diagnosis (years)	Mutation identified by targeted sequencing in present or previous study? [(1-3)]	Target seq or WGS in present study?
H7T	-	-	H7T	FFPE	28	None	Target[(1)]
H15T	-	-	H15T	FFPE	30	None	Target[(1)]
SS_53	-	-	-	FFPE	33	None	Target[(1)]
H10T	-	-	H10T	FFPE	36	None	Target[(1)]
SS22	-	SS22	-	FFPE	41	None	Target[(1)]
H16T	-	-	H16T	FFPE	43	None	Target[(1)]
SS_49	-	-	-	FFPE	47	None	Target[(1)]
H17T	-	-	H17T	FFPE	47	None	Target[(1)]
SS37	-	SS37	-	FFPE	47	None	Target[(1)]
H6T	SS3	-	H6T	Frozen	48	None	Target[(1)]
H9T	-	-	H9T	FFPE	48	None	Target[(1)]
SS_46	-	-	-	FFPE	50	None	Target[(1)]
SS7	-	SS7	-	FFPE	50	None	Target[(1)]
SS7	-	SS7	-	FFPE	52	None	Target[(1)]
H13T	-	-	H13T	FFPE	53	None	Target[(1)]
H8T	-	-	H8T	FFPE	55	**NRAS c.182A>G (p.Q61R)[(1)]**	Target[(1)]
SS_50	-	-	-	FFPE	62	None	Target[(1)]
H19T	-	-	H19T	FFPE	62	None	Target[(1)]
SS11	-	SS11	-	FFPE	64	None	Target[(1)]
SS_51	-	-	-	FFPE	65	None	Target[(1)]
SS2	-	SS2	-	FFPE	65.4	None	Target[(1)]
H5T	-	-	H5T	FFPE	70	None	Target[(1)]
H20T	-	-	H20T	FFPE	70	None	Target[(1)]
SS_14	-	-	-	FFPE	71	None	Target[(1)]
H14T	-	-	H14T	FFPE	72	None	Target[(1)]
SS4	-	SS4	-	FFPE	75	None	Target[(1)]
SS5	-	SS5	-	FFPE	76	None	Target[(1)]
SS8	-	SS8	-	FFPE	86	**NRAS c.182A>G (p.Q61R)[(1)]**	Target[(1)]
SS1	-	SS1	-	FFPE	86.5	None	Target[(1)]
SpT1	SS1	-	H1T	Frozen	44	None	**WGS**
SpT3	SS5	-	H2T	Frozen	66	None	**WGS**
SpT4	SS4	-	H3T	Frozen	48	None	**WGS**
SpT6	-	-	H11T	Frozen	49	None	**WGS**
SpT8	-	-	H12T	Frozen	60	None	**WGS**

(1) Hotspot regions of seven genes (*FGFR2, FGFR3, HRAS, KRAS, PTPN11, NRAS, RET*) were screened using molecular inversion (MIP) probes and next-generation (IonTorrent) sequencing in the present study. For details of regions screened, see S6A Table.

(2) In this 2009 study, a total of 30 FFPE SpT samples were screened using PCR and dideoxy-sequencing for mutation hotspots in 17 genes (*AKT1, BRAF, CTNNB1, EGFR, FGFR1, FGFR2, FGFR3, HRAS, KIT, KRAS, MAP2K1, MAP2K2, NRAS, PIK3CA, PTPN11, RET* and *SOS1*). Age information at time of diagnosis was available for 28 patients. Of these 30 samples, ten mutation-negative tumours, for which we had sufficient amount of DNA, were re-run in the present study. Note that the SS8 sample in which we identified a *NRAS* mutation in the present study had a very low PCR amplification success rate in the 2009 study (<30%).

(3) In this 2013 study, DNA from 33 SpT (FFPE and frozen) samples was PCR amplified and screened for hotspot mutations in *FGFR3* and *HRAS* only by dideoxy-sequencing. Age information at the time of diagnosis was available for 26 patients. In the present study, 18 mutation-negative samples, which had sufficient amount of DNA of good quality, have been re-run for the seven gene panel.
