# Supplementary material for: Whole-genome sequencing of spermatocytic tumors provides insights into the mutational processes operating in the male germline
Source: PLoS One. 2017 May 22;12(5):e0178169. doi: 10.1371/journal.pone.0178169 (PMC5439955; doi:10.1371/journal.pone.0178169)
Supplement: S5 Table — (PDF) [file pone.0178169.s008.pdf]

S5 Table - Summary of the ENCODE methylation dataset for 3 tissue samples or cell lines and comparison with SpT mutations.

**Samples**	**BC_Testis_N30**	**GM12878**	**H1-hESC**
Sites in both replicates	1151596	1048775	1118911
Number of sites with >50% reads methylated	266890	217632	320806
Proportion of sites with >50% reads methylated	23.17%	20.75%	28.67%
Overlap with SpT mutations	11	7	9
Overlapping SpT mutations >50% methylated	10	4	9
Binomial p value	3.87E-06	0.03783	1.31E-05
